# Mephedrone in Adolescent Rats: Residual Memory Impairment and Acute but Not Lasting 5-HT Depletion

**DOI:** 10.1371/journal.pone.0045473

**Published:** 2012-09-18

**Authors:** Craig P. Motbey, Emily Karanges, Kong M. Li, Shane Wilkinson, Adam R. Winstock, John Ramsay, Callum Hicks, Michael D. Kendig, Naomi Wyatt, Paul D. Callaghan, Iain S. McGregor

**Affiliations:** 1 School of Psychology, University of Sydney, New South Wales, Australia; 2 Department of Pharmacology, University of Sydney, New South Wales, Australia; 3 School of Chemistry, University of Sydney, New South Wales, Australia; 4 Institute of Psychiatry, Kings College, University of London, United Kingdom; 5 TICTAC Communications Ltd., St George’s College, University of London, United Kingdom; 6 Life Sciences Division, Australian Nuclear Science and Technology Organisation, Sydney, New South Wales, Australia; Sapienza University of Rome, Italy

## Abstract

Mephedrone (4-methylmethcathinone, MMC) is a popular recreational drug, yet its potential harms are yet to be fully established. The current study examined the impact of single or repeated MMC exposure on various neurochemical and behavioral measures in rats. In Experiment 1 male adolescent Wistar rats received single or repeated (once a day for 10 days) injections of MMC (30 mg/kg) or the comparator drug methamphetamine (METH, 2.5 mg/kg). Both MMC and METH caused robust hyperactivity in the 1 h following injection although this effect did not tend to sensitize with repeated treatment. Striatal dopamine (DA) levels were increased 1 h following either METH or MMC while striatal and hippocampal serotonin (5-HT) levels were decreased 1 h following MMC but not METH. MMC caused greater increases in 5-HT metabolism and greater reductions in DA metabolism in rats that had been previously exposed to MMC. Autoradiographic analysis showed no signs of neuroinflammation ([^125^I]CLINDE ligand used as a marker for translocator protein (TSPO) expression) with repeated exposure to either MMC or METH. In Experiment 2, rats received repeated MMC (7.5, 15 or 30 mg/kg once a day for 10 days) and were examined for residual behavioral effects following treatment. Repeated high (30 mg/kg) dose MMC produced impaired novel object recognition 5 weeks after drug treatment. However, no residual changes in 5-HT or DA tissue levels were observed at 7 weeks post-treatment. Overall these results show that MMC causes acute but not lasting changes in DA and 5-HT tissue concentrations. MMC can also cause long-term memory impairment. Future studies of cognitive function in MMC users are clearly warranted.

## Introduction

Mephedrone (“Meow”, 4-methylmethcathinone, MMC) is a substituted cathinone that has rapidly gained popularity over recent years as a recreational drug [1], [2], [3]. The cathinones display pharmacological properties that closely resemble slightly less potent equivalents of their amphetamine analogs [4]. Users of MMC report effects that are reminiscent of methamphetamine (METH: stimulant, euphoric), cocaine (“moreish”, propensity to cause binges) and 3,4-methylenedioxymethamphetamine (MDMA: entactogenic, prosocial) [5], [6], [7]. Studies of MMC are at a formative stage with the neurochemical actions and toxicity underexplored relative to the widespread use of the drug.

Our group recently characterized the activational “signature” of MMC (30 mg/kg) in the brain relative to METH (2 mg/kg) in adolescent rats using Fos immunohistochemistry [8]. Consistent with the subjective reports of users, the Fos “signature” of MMC resembled a combination of METH and MDMA. This analysis is also supported by recent microdialysis results indicating that low doses of MMC (1–3 mg/kg) cause a robust amphetamine-like increase in dopamine (DA) and a profound MDMA-like increase in 5-hydroxytryptamine (5-HT) in the nucleus accumbens [9].

Consistent with these observations, recent *in vitro* studies indicate that MMC has affinity for both the 5-HT transporter (SERT) and DA transporter (DAT) and inhibits striatal synaptosomal DA uptake (like METH) and hippocampal synaptosomal 5-HT uptake (like MDMA) [10], [11], [12]. A substantial serotonergic effect of MMC is also indicated by clinical evidence of the “serotonin syndrome” in some MMC users [13].

Overall, there is only sparse evidence regarding the possible harms arising from MMC use. Clinical reports suggest users occasionally suffer from agitation, tachycardia, hypertension and seizures [14], [15], [16], [17], [18]. However, in almost all reports, sedation and symptomatic treatment allows patients to be discharged in a fairly short time (typically within hours) without obvious lasting ill effects. While a number of deaths have been linked to MMC [18], [19], [20], [21], [22], there are few in which a causal role for MMC has been unequivocal.

Information on long-term harms is largely limited to anecdotal reports of cognitive impairment and mood disturbance in on-line discussion sites such as *Drugs-Forum*, *Bluelight* and *Erowid*. Although only a handful of peer-reviewed investigations into potential MMC-induced harms have been published to date, these have largely failed to demonstrate lasting impairment. MMC does not appear to cause dopaminergic neurotoxicity in mice [23], nor does it induce a lasting impairment of cardiac function in rats [24]. Rats given moderate doses of MMC (3×10 mg/kg, S.C., once every 2 h) showed no alteration in levels of cortical or striatal noradrenaline, DA or 5-HT when measured two weeks later [12].

However, two recent publications do give some cause for concern. Repeated high doses of MMC (4×10 or 4×25 mg/kg, I.P., once every 2 h), paired with elevated ambient temperatures (≥27°C), depleted 5-HT when measured 7 days later in rats [10]. This is an effect reminiscent of that seen with high doses of MDMA in laboratory animals [25], [26], [27], [28], although controversy continues as to the significance of such effects for humans [12], [29]. It is unknown whether the 5-HT depletion reported in rats following binge dosing with MMC is permanent. Secondly, recently published work by Freeman et al. [30] is suggestive of lasting cognitive impairment (as measured by a prose recall task) in human MMC users.

In the present study we therefore sought to clarify whether lasting decreases in regional levels of 5-HT or DA occurred following exposure to MMC in rats and to investigate the possibility that this treatment may induce lasting cognitive impairment. Our previous research has documented cognitive and social impairment in rats weeks or months after relatively brief exposure to drugs such as MDMA and METH [25], [27], [28], [31], [32], [33]. These effects are consistent with reports from other groups [34], [35], [36], [37].

Autoradiographic analysis was also performed in the present study. The ligand [^125^I]CLINDE [38], [39] was used to quantify levels of translocator protein (TSPO) in rats repeatedly exposed to MMC or METH. TSPO receptors display upregulation with neurodegeneration, concurrent with the microglial activation observed during neuroinflammatory processes [40]. As inflammatory responses may play an important role in the toxic potential of several drugs of abuse [40], [41], [42], [43], it was of interest to test whether MMC exposure induced this response.

Finally, it was of interest to determine whether repeated exposure to MMC causes sensitization, an effect sometimes reported with MDMA and METH, whereby the neurochemical and behavioral response to the drug is augmented with repeated administration [44], [45], [46]. We therefore compared the acute behavioral (locomotor hyperactivity) and neurochemical (regional DA and 5-HT) response to MMC in rats receiving their first or tenth dose of the drug.

## Experimental Procedures

### Subjects

Experiment 1 used 40 adolescent male Wistar rats (aged 3–4 weeks at delivery to our facility; mean weight on delivery 113 g) obtained from the University of Adelaide. Experiment 2 used 48 adolescent male Wistar rats (aged approximately 3 weeks at delivery; mean weight on delivery 126 g) sourced from the Animal Resources Centre (Perth, Western Australia). Rats were housed in groups of eight in large plastic tubs in a temperature-controlled colony room (22±2°C) on a 12 h light/dark cycle (lights on from 8∶00 PM to 8∶00 AM). Rats were acclimatized to their new surroundings for several days before commencing a program of handling and habituation. They were provided with food and water *ad libitum*. All behavioral testing occurred during the dark cycle. All efforts were made to minimize both the number of rats used and distress to the rats involved. All experimentation was approved by the University of Sydney Animal Ethics Committee (ethics protocol L29/5-2010/3/5330).

### Drugs and Doses

MMC was obtained from the U.K. Customs Service, sourced from seizures made shortly after the banning of MMC in that country (April 2010). The customs-sourced MMC was analysed by High Performance Liquid Chromatography (HPLC) performed on a Waters 2695 Separations module equipped with the Waters Alliance Series Column Heater (set at 32°C) and Waters 2996 Photodiode Array (PDA) Detector. Samples were resolved on a Waters XBridge C18 5 µm column (2.1×150 mm) using an isocratic flow of 30 v/v% aqueous acetonitrile with 0.1 v/v% triethylamine at a flowrate of 0.2 mL/min. Chromatograms were extrapolated at a UV maximum of 258 nm with data acquisition and processing performed with the Waters Empower 2 software. Samples were analysed in triplicate.

Reference-grade METH was obtained from the National Measurement Institute (New South Wales, Australia). All drugs were dissolved in physiological saline and injected I.P. at a volume of 1 ml/kg. The dose of MMC used in Experiment 1 (30 mg/kg) was taken from our recent study [8] in which it produced robust hyperactivity and brain activation without any apparent adverse consequences to the health of the rats. The dose of METH selected (2.5 mg/kg) was slightly higher than was used in this earlier study (2 mg/kg) as we found METH hyperactivity at 2 mg/kg in adolescent rats to be considerably below that seen with MMC (30 mg/kg) [8].

In Experiment 2 a range of MMC doses were utilized (7.5, 15 and 30 mg/kg, injected I.P. once every day for 10 days) to determine possible long-term adverse consequences of repeated lower doses of MMC, similar to those we have found with relatively low doses of MDMA [27], [28], [33].

### Experiment 1: Effects of Acute and Repeated METH and MMC on Locomotor Activity, DA and 5-HT

#### Procedure

Five days after delivery rats were divided into five groups (VEH, METH×1, METH×10, MMC×1, MMC×10) with n = 8 per group. All rats were injected once per day for 10 consecutive days. VEH animals received 0.9% saline injections each day while METH×1 and MMC×1 rats received saline injections for the first 9 days and an injection of METH (2.5 mg/kg) or MMC (30 mg/kg) respectively on the 10th day. METH×10 and MMC×10 animals received injections of METH (2.5 mg/kg) or MMC (30 mg/kg) respectively on each of the 10 days.

On the first and final test days, individual rats were removed from their home cage and injected before being immediately placed into locomotor testing chambers. Rats had been previously acclimatized to the test chambers for 2 days prior to the day 1 test. On each of these days, rats were treated identically to the first dosing day except that they received vehicle injections rather than drug doses. The order of testing was counterbalanced across treatment conditions in order to control for time of day effects. Ambient temperature was approximately 26°C throughout the locomotor activity testing sessions. At 1 h after their final drug treatment rats were rapidly decapitated and their brains removed. HPLC analysis of striatal and hippocampal neurotransmitters was carried out as described below.

#### Apparatus

Locomotor activity was assessed in dark enclosed chambers (60 cm length × 26 cm depth × 33 cm height) with red perspex lids located in a room dimly illuminated by a 40 W red bulb. The floor of each chamber consisted of metal rods and the chambers were fitted with a miniature overhead infrared video camera connected to a PC that used automated video tracking software (TRACKMATE 1.0, Motion Mensura, Cooks Hill, NSW, Australia) to determine the locomotor activity (distance travelled) of the rat.

#### Neurotransmitter and metabolite analysis

HPLC methods were as described previously [27]. Brains were first sagitally bisected, with one hemisphere set aside for autoradiography analysis. The remaining hemispheres were manually dissected over dry ice into five regions of interest and snap frozen in liquid nitrogen, and then stored at −80°C until analysis. Tissue samples from striatum and hippocampus were weighed and homogenized in 500 µl of 0.2 M perchloric acid containing 0.1% cysteine and 400 nM of internal standard (5-hydroxy-N-methyltryptamine; 5-HMeT) using a glass-Teflon homogenizer and Brinkman polytron. The homogenate was then centrifuged for 15 min at 15,000 g, 4°C and pellet discarded. A 10 µl aliquot was then analysed for bioamine content using HPLC with electrochemical detection.

Briefly, the HPLC system consisted of a Shimadzu ADvp module (Kyoto, Japan) equipped with SIL-10 autoinjector with sample cooler and LC-10 on-line vacuum degassing solvent delivery unit. Chromatographic control, data collection and processing were carried out using Shimadzu Class VP data software. The mobile phase consisted of 0.1 mol/l phosphate buffer (pH 3.0), PIC B-8 octane sulphonic acid (0.74 mmol/l; Waters, Australia), sodium EDTA (0.3 mmol/l) and methanol (18% v/v). The flow rate was maintained at 0.7 ml/min.

Chromatographic separation of DA, dihydroxyphenylacetic acid (DOPAC), homovanillic acid (HVA), 5-hydroxyindolacetic acid (5-HIAA) and 5-HT were accomplished on a Phenomenex Synergi Polar-RP 80A (250×4.6, 4 µm) column maintained at 30°C. Quantification was achieved via an INTRO electrochemical detector (Antec Leyden, Netherlands) equipped with Faraday-shielded oven compartment and a glassy carbon working electrode set at +0.75 V. The concentrations of unknown samples were obtained from the linear regression equation of calibration curves by plotting concentration versus area ratio of the external standard and internal standard. HVA+DOPAC/DA ratios were used as an index of DA metabolism, while 5-HIAA/5-HT ratios were used for 5-HT metabolism.

#### Autoradiography

Autoradiographic analysis was only conducted on the repeatedly dosed rats in Experiment 1. Due to the exploratory nature of the study and the expense of autoradiographic techniques the analysis was limited to those experimental conditions most likely to produce a clear result (i.e. maximal doses and shortest time between dosing and analysis).

Brains were rapidly removed and one hemisphere was dissected for HPLC analysis (see above) while the other was snap-frozen whole in liquid nitrogen and stored at −80°C. Sagittal sections (20 µm) of the intact hemisphere were cut on a cryostat, and mounted onto polysine-coated slides (LabServ, Australia). [^125^I]CLINDE was used to assess binding density for TSPO as described previously [38]. TSPO binding was assessed in the hippocampus, substantia nigra, anterior thalamus, olfactory tubercle, striatum and nucleus accumbens.

Air dried slides were either apposed against Kodak Biomax MR film, developed, and films acquired through a BioRad GS-800 densitometer, or apposed against FujiFilm SR phosphorimaging plates and read in a FujiFilm FLA7000 phosphorimager system. Images were analysed using ImageJ software (NIH, USA). Standard curves were calculated for conversion of optical density to nCi/mg radioactivity concentration using calibrated ^14^C microscales (American Radiochemical Company, USA).

### Experiment 2: Long-term Residual Effects of MMC

Experiment 2 involved 4 groups of rats (VEH, MMC7.5, MMC15, MMC30) with n = 12 per group. Beginning 4 days after delivery to the colony (age 22–27 days) all rats were dosed once per day for ten consecutive days with either vehicle or MMC (7.5, 15 or 30 mg/kg). Dosing was conducted in the colony room, with all rats being weighed immediately prior to dosing. Following injection they were returned to their home cages. The sequence of behavioral testing used in this experiment to assess residual effects of MMC dosing is shown in Table 1.

**Table 1:** The test sequence employed in Experiment 2.

Experiment Day	1	2	3	4	5	6	7	8	9	10	21	25	45	57
Dosing	√	√	√	√	√	√	√	√	√	√				
Elevated Plus Maze											√			
Social Preference												√		
Novel Object Recognition													√	
Euthanasia														√

#### Behavioral Tests

##### Elevated Plus Maze

This test was conducted 11 days after the conclusion of dosing. As described previously [47] the apparatus consisted of a red perspex maze (floored with black laminate) with two open arms (50 × 10 cm) and two opposite closed arms (50 × 10 cm) that had 40 cm high walls. The open and closed arms were connected by a central square (10 × 10 cm). The maze was elevated to a height of 70 cm. The testing room was illuminated by a red light (40 W). Rats were placed in the centre of the apparatus, with their head facing a closed arm. Testing continued for 5 min during which time spent on the open and closed arms and the distance travelled was assessed using automated video tracking software (TRACKMATE 1.0, Motion Mensura, Cook’s Hill, NSW, Australia). In between each test session the maze was thoroughly wiped down with a damp cloth containing an ethanol solution.

##### Social Preference

Two weeks after the conclusion of dosing, rats were tested in a social preference test as described previously [8]. The social preference test apparatus consisted of a wooden arena (1200 mm long × 1200 mm wide × 830 mm high) in a room dimly illuminated by a 40 W red bulb. This enclosure was fitted with a mesh grid floor and both walls and floor were painted matt black. Animal behavior was recorded as in the Elevated Plus Maze test above.

The social preference arena contained two small cages on opposite walls (300 mm wide × 200 mm long × 200 mm deep). One cage contained an adolescent Wistar rat that was of similar age and weight to the test subject, but unfamiliar to the test animal. A new caged animal was used for each test. The other cage contained a life-sized toy rat; this was included to control for the possibility of the subject rat visiting the caged rat purely out of visual curiosity. Time spent within close proximity (150 mm) of the caged rat was taken as an index of social motivation [48], [49]. Social preference and locomotor activity was measured for 10 min before rats were returned to their home cages.

##### Novel Object Recognition

Novel object recognition (NOR) testing took place 35 days after the conclusion of dosing, in an opaque black plastic circular arena (80 cm diameter × 51 cm height). Rats were given repeated habituation exposures to the testing arena for several days prior to training in the absence of any objects. The following day rats received a paired trial consisting of a sample trial and a test trial. During the sample trial rats were individually placed in the arena for 3 min in the presence of two identical objects, spaced 15 cm from the periphery on opposite sides of the arena. The rat was then returned to a holding cage, and after a 15 min delay the test trial was conducted. During the test trial the rat was placed in the arena for 3 min with one object the same as in the sample trial and a new, “novel” object that was different in shape, size and texture. All objects were fixed to the floor of the arena or heavily weighted so they could not be moved by the rats. Between each trial, the arena and objects were cleaned with 70% ethanol solution and air-dried, and triplicate sets of all objects were used to ensure no odors were present or associated with any of the objects or arena. The objects were a plastic spray bottle and a ceramic oil burner, all painted matt black. Test objects, placement of the novel object and order of testing were counterbalanced across conditions.

Scoring of object investigation was conducted from video (using OD Log software: www.macropodsoftware.com) by an observer who was blind as to experimental condition and the identity of the novel object. Only active investigation (i.e. sniffing, pawing, biting) was counted as investigation; perching on top of the object was not counted as investigation.

#### Neurotransmitter and metabolite analysis

At 47 days after the conclusion of drug treatment rats were rapidly decapitated and their brains removed. HPLC analysis was carried out as per Experiment 1.

### Data Analysis

In Experiment 1, behavioral measures and HPLC results were compared across the different treatment conditions using planned contrasts with Bonferroni adjustments. The critical comparisons were as follows:

1. VEH *versus* METH (both METH×1 and METH×10 groups), which allows determination of overall METH effects;
2. VEH *versus* MMC (both MMC×1 and MMC×10 groups), which allows determination of overall MMC effects;
3. METH×1 *versus* METH×10, which allows determination of possible METH sensitization effects;
4. MMC×1 *versus* MMC×10, which allows determination of possible MMC sensitization effects.

A significance level of P<0.0125 was used which represents the alpha level (0.05) divided by the number of contrasts (4, Bonferroni correction). For the autoradiography analysis, only the first two contrasts were required (due to the exclusion of the singly-dosed groups). These contrasts therefore used a significance level of P<0.025 (Bonferroni correction). For Experiment 2, a simple one-way ANOVA followed by post hoc Dunnett’s test was used to compare each MMC group (MMC7.5, MMC15, and MMC30) with the control (VEH) group on key behavioral and neurochemical measures.

In both experiments the Levene test was employed to ensure homogeneity of variance between groups. When this was not satisfied a logarithmic or square root transformation of the data was applied (in all cases, the transformation with a higher *P* value on Levene’s test was used).

## Results

### Drug Analysis

HPLC analysis of the customs-sourced MMC utilized in both experiments found it to have a purity of 99.65±0.09% (t_R_ 10.4 min, ë_max_ 258 nm).

### Experiment 1

#### Weight gain

**Figure 1:**
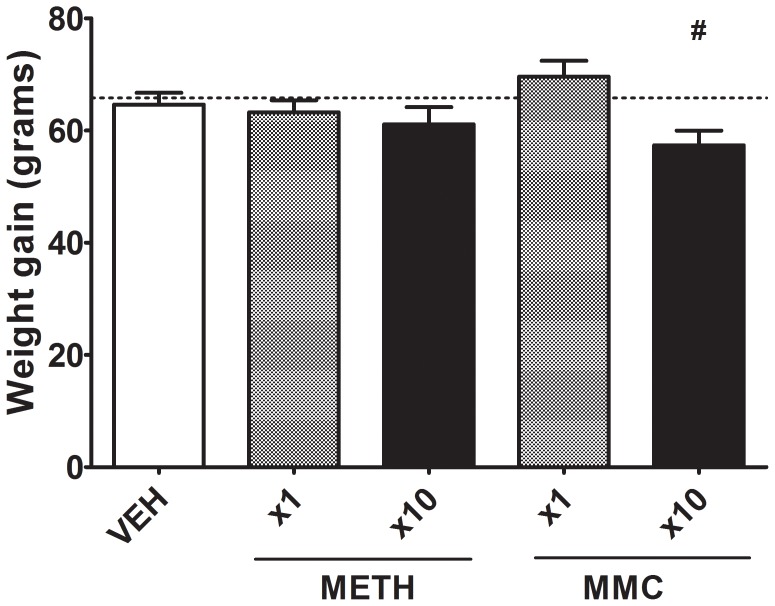
Weight gain over the 10 dosing days. Adolescent rats were given either saline injections, MMC (30 mg/kg/day) or METH (2.5 mg/kg/day) once per day during this period. # represents a significant difference when comparing the MMC×10 group with the combined VEH, METH×1 and MMC×1 groups (the latter groups had not received their single dose of the drug at the time when the weights were measured). The dotted line represents the mean for the combined VEH, METH×1 and MMC×1 groups.

Weight gain measurements over the 10 day injection period (Figure 1) showed that the MMC×10 group gained significantly less weight than a composite control group containing all of the rats that received vehicle treatment over the first 9 days of the experiment (VEH group, MMC×1 group and METH×1 group). This composite control group was appropriate given that the MMC×1 and METH×1 group had not received any drug at the beginning of day 10 of dosing when these weight measures were taken. No other comparisons were significant.

#### Locomotor Activity

**Figure 2:**
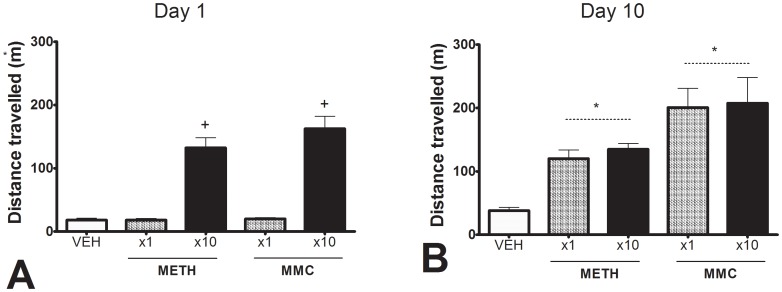
Distance travelled. Locomotor activity in the hour following dosing in rats given their first or tenth daily dose of methamphetamine (METH, 2.5 mg/kg) or mephedrone (MMC, 30 mg/kg). Error bars represent + S.E.M. On the first day (Figure 2A), the two drug treated groups (MMC×10 and METH×10) travelled greater distance than the three vehicle-treated groups (P<0.01, indicated by ++). On day 10 (Figure 2B) both the combined METH groups and the combined MMC groups travelled greater distance than the VEH group (P<0.01, indicated by *). A logarithmic transformation was applied to the data due to heterogeneity of variance.

The results for distance travelled in the hour following dosing on day 1 and day 10 are presented in Figure 2. The locomotor response of the MMC and METH groups significantly exceeded that of the VEH group (Overall ANOVA F_4,35_ = 22.72, P<0.001; both contrasts P<0.01). For both METH and MMC, the ×10 groups did not significantly differ from the ×1 groups, suggesting that little if any locomotor sensitization had developed.

#### Neurotransmitter Levels

HPLC results showing neurotransmitter and metabolite levels 60 min after the 10^th^ day of dosing in Experiment 1 are presented in Table 2.

**Table 2:** Mean (SEM) neurotransmitter and metabolite levels in brains collected 1 h post-mortem during Experiment 1.

	VEH	METH×1	METH×10	MMC×1	MMC×10
***Striatum***					
Dopamine	4690 (313)	5985 (445)^1^	6766 (269)^1^	6753 (621)^2^	5825 (479)^2^
HVA	588 (39)	538 (38)	646 (41)	674 (67)^3^	453 (45)^3^
DOPAC	665 (28)	612 (43)	680 (51)	635 (38)	492 (50)
HVA+DOPAC/DA	0.27 (0.02)	0.19 (0.01)^1^	0.20 (0.01)^1^	0.20 (0.01)^2^	0.16 (0.01)^2^
5-HT	305 (23)	338 (30)	359 (17)	218 (14)^2^	203 (23)^2^
5-HIAA	719 (46)	798 (61)	812 (38)	747 (46)	594 (38)
5-HIAA/5-HT H	2.38 (0.10)	2.38 (0.06)	2.27 (0.04)	3.43 (0.17)^2 3^	3.02 (0.10)^2 3^
***Hippocampus***					
5-HT	99 (7)	101 (5)	104 (5)	52 (5)^2^	53 (4)^2^
5-HIAA	172 (9)	197 (7)	190 (9)	155 (7)	141 (9)
5-HIAA/5-HT H	1.69 (0.11)	1.93 (0.05)	1.85 (0.07)	3.06 (0.17)^2 3^	2.67 (0.08)^2 3^

Data are mean (S.E.M.). Neurotransmitter and metabolite levels are expressed in ng/g wet tissue, metabolism measures are expressed as ratios. VEH = vehicle injections only; METH×1 = 2.5 mg/kg methamphetamine I.P. once; METH×10 = 2.5 mg/kg methamphetamine I.P. once/day for ten days; MMC×1 = 30 mg/kg mephedrone I.P. once; MMC×10 = 30 mg/kg mephedrone I.P. once/day for ten days.

H indicates heterogeneity of variance in untransformed data.

1 sig. diff. (P<0.01) comparing VEH to combined METH groups;

2 sig. diff. (P<0.01) comparing VEH to combined MMC groups;

3 sig. diff. (P<0.01) comparing MMC×1 to MMC×10 groups.

Both the combined MMC and the combined METH groups had significantly elevated levels of striatal DA relative to VEH (Overall ANOVA F_4,31_ = 3.36, P<0.05; both significant contrasts P<0.01). No other contrasts were significant for DA.

Striatal levels of HVA in the MMC×1 group significantly exceeded those of the MMC×10 group (Overall ANOVA F_4,31_ = 3.49, P<0.05; MMC×1 vs MMC×10 contrast P<0.01). No other contrasts were significant for HVA. For DOPAC there was an overall significant ANOVA (F_4,31_ = 2.77, P<0.05). There was a non-significant trend towards depression of striatal DOPAC in the MMC×10 group relative to MMC×1, an effect that did not survive Bonferroni correction (P = 0.032, n.s.).

Striatal DA metabolism ((HVA + DOPAC)/DA) was significantly reduced relative to VEH for both METH and MMC groups (Overall ANOVA F_4,31_ = 10.78, P<0.001; both contrasts P<0.001). No other contrasts were significant on this measure, although again there was again a strong trend towards a greater depression of striatal DA metabolism in the MMC×10 group relative to MMC×1 group (P = 0.033, n.s.).

MMC treated rats displayed significantly diminished levels of striatal 5-HT relative to VEH (Overall ANOVA F_4,31_ = 10.18, P<0.001; contrast P<0.01). No other contrasts were significant on this measure. No significant differences were found with striatal 5-HIAA levels, although there was again a non-significant trend towards 5-HIAA depression in the MMC×10 group relative to the MMC×1 group (Overall ANOVA F_4,31_ = 3.43, P<0.05; contrast P = 0.03, n.s.).

Striatal 5-HT metabolism (5-HIAA/5-HT) was significantly increased in MMC groups relative to VEH (Overall ANOVA F_4,31_ = 26.74, P<0.001; contrast P<0.001). 5-HT metabolism was also significantly elevated in the MMC×1 group relative to the MMC×10 group (P<0.01). No other contrasts were significant.

In the hippocampus, as with the striatum, 5-HT levels were significantly lower in MMC groups compared to VEH (Overall ANOVA F_4,29_ = 30.35, P<0.001; contrast P<0.001); no other hippocampal 5-HT contrasts were significant. No contrasts proved significant on hippocampal 5-HIAA levels, although there was a non-significant trend towards elevated hippocampal 5-HIAA levels in METH animals relative to VEH (Overall ANOVA F_4,29_ = 8.74, P<0.001; contrast P<0.014, n.s.). Hippocampal 5-HT metabolism was significantly elevated in MMC groups relative to VEH (Overall ANOVA F_4,29_ = 32.88, P<0.001; contrast P<0.001). A non-significant trend was apparent towards elevated hippocampal 5-HT metabolism in the MMC×1 group relative to the MMC×10 group (p = 0.04, n.s.).

#### Autoradiography Results

Results of the autoradiographic analyses are presented in Table 3. No significant differences were found in CLINDE binding in any region examined.

**Table 3:** Autoradiographic analysis of [^125^I]CLINDE binding in repeatedly-dosed animals compared to control.

	VEH	METH×10	MMC×10
Anterior Thalamus	11.89 (1.01)	9.52 (1.77)	10.61 (1.57)
Hippocampus	10.85 (0.86)	8.54 (1.52)	9.68 (0.79)
Nucleus Accumbens	9.57 (1.02)	6.16 (1.09)	7.76 (1.04)
Olfactory Tubercle	9.40 (1.50)	9.26 (1.58)	9.25 (1.05)
Striatum	9.08 (0.93)	7.49 (1.55)	11.47 (1.33)
Substantia Nigra	15.67 (1.92)	16.18 (2.85)	11.56 (2.45)

Data are mean (S.E.M.). Values are nCi/mg specific binding of [^125^I]CLINDE. VEH = vehicle injections only; METH×10 = 2.5 mg/kg methamphetamine I.P. once/day for ten days; MMC×10 = 30 mg/kg mephedrone I.P. once/day for ten days. Brain tissue harvested 1h after final dose.

### Experiment 2

#### Long-term residual effects of MMC

**Figure 3:**
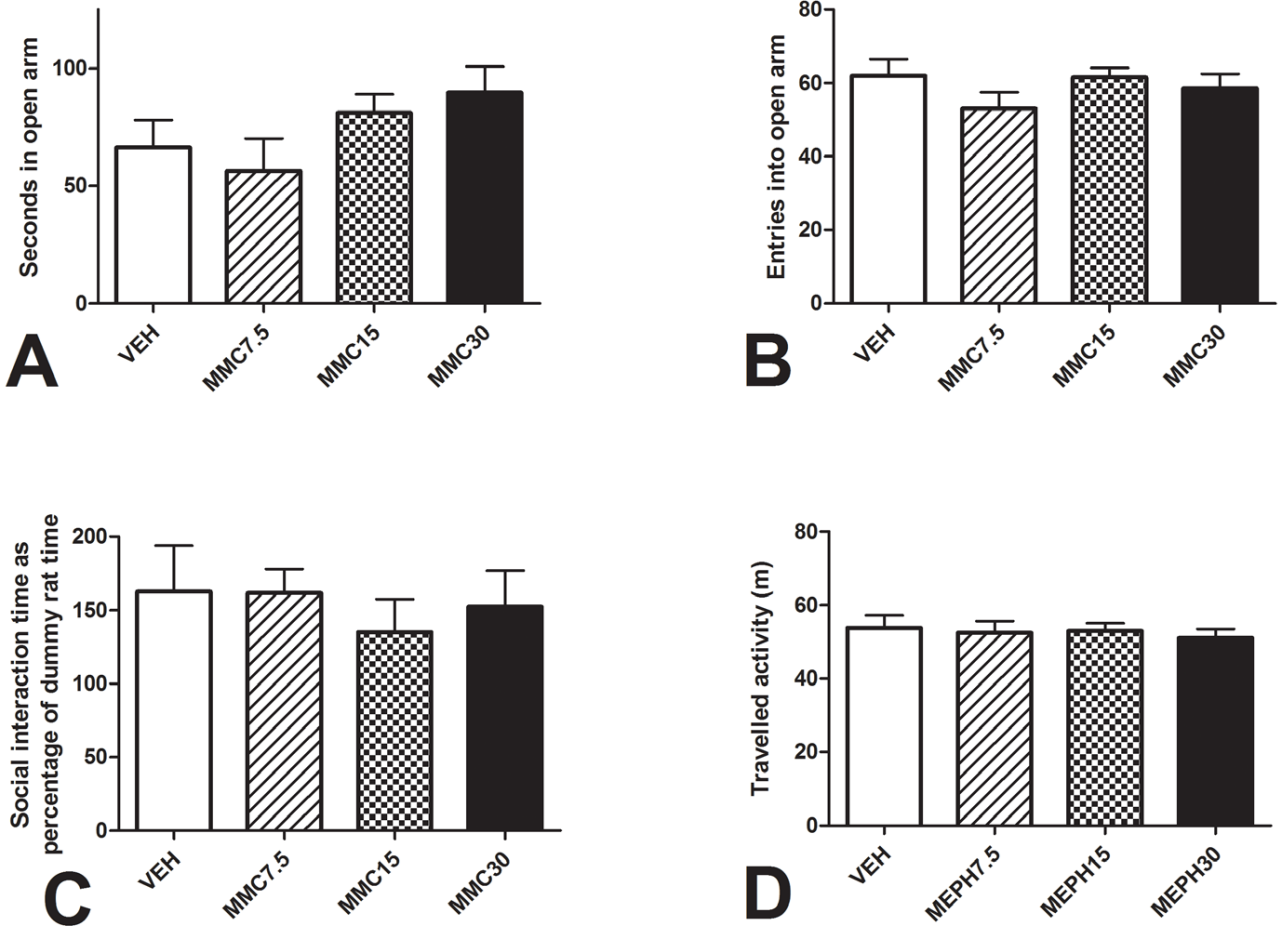
Residual MMC effects. **A.** Open arm time, and **B.** Distance travelled on the Elevated Plus Maze which was run 11 days after the conclusion of dosing. **C.** Investigation time for the fake rat and the real rat, and **D.** Distance travelled in the Social Preference test which was run 15 days after the conclusion of dosing. Error bars represent + S.E.M. No significant differences were found between groups on either test. VEH = saline 1 ml/kg I.P., MMC7.5 = 7.5 mg/kg mephedrone I.P., MMC15 = 15 mg/kg mephedrone I.P., MMC30 = 30 mg/kg mephedrone I.P.

In Experiment 2, none of MMC-treated groups significantly differed from the VEH group in weight gain either during dosing (F_3,37_ = 0.29, P = 0.83) or during the post-dosing period (F_3,37_ = 0.33, P = 0.80). None of the MMC-treated groups differed from the VEH group on the elevated plus maze or social preference tests (results summarized in Figure 3).

**Figure 4:**
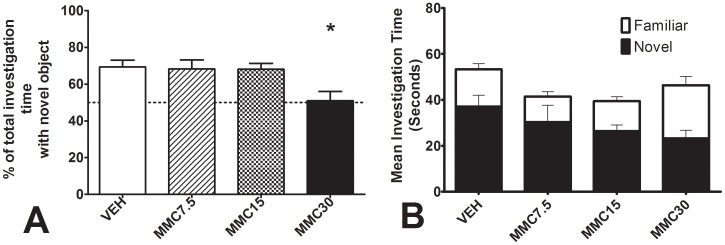
Performance on the Novel Object Recognition test. A. Percentage of total investigation time spent investigating the novel object (dotted line represents chance performance). **B.** Time spent inspecting familiar and novel objects. Test was conducted 35 days after the conclusion of dosing and 15 min after a 3 min training exposure. See text for further details. Error bars represent + S.E.M.; ** represents significant difference from VEH (Dunnett’s test, P<0.01). VEH = saline 1 ml/kg I.P., MMC7.5 = 7.5 mg/kg mephedrone I.P., MMC15 = 15 mg/kg mephedrone I.P., MMC30 = 30 mg/kg mephedrone I.P.

Significant differences between groups were obtained on the novel object recognition test (Figure 4), with the MMC30 group alone showing significantly impaired memory relative to the VEH group (Overall ANOVA on preference for novel object F_3,43_ = 4.43, P<0.01; Dunnet’s contrast on MMC30 against VEH P<0.05). None of the MMC-treated groups differed from the VEH group in total inspection time during the novel objection recognition test (F_3,43_ = 0.86, P = 0.47).

#### Neurotransmitter Levels

HPLC results from Experiment 2 are presented in Table 4. None of the MMC-treated groups differed from the VEH group in DA and 5-HT levels or in 5-HIAA/5-HT or HVA+DOPAC/DA ratios in the striatum or hippocampus.

**Table 4:** Mean (S.E.M.) neurotransmitter and metabolite levels 47 days after final dose.

	VEH	MMC(7.5)	MMC(15)	MMC(30)
***Striatum***				
Dopamine	8256 (1006)	8731 (1239)	9067 (1082)	8487 (621)
DOPAC	827 (98)	802 (95)	855 (115)	824 (77)
HVA	678 (85)	674 (86)	689 (93)	658 (57)
HVA+DOPAC/DA	0.18 (0.01)	0.18 (0.16)	0.17 (0.16)	0.18 (0.16)
5-HT	301 (31)	303 (37)	377 (48)	300 (21)
5-HIAA	477 (56)	464 (58)	529 (58)	473 (38)
5-HIAA/5-HT	1.57 (0.05)	1.54 (0.04)	1.43 (0.04)	1.58 (0.06)
***Hippocampus***				
5-HT	140 (8)	127 (6)	125 (9)	130 (5)
5-HIAA	209 (9)	210 (14)	193 (12)	208 (12)
5-HIAA/5-HT	1.51 (0.06)	1.67 (0.14)	1.62 (0.16)	1.62 (0.11)

Data are mean (S.E.M.). Neurotransmitter and metabolite levels are expressed in ng/g wet tissue, metabolism measures are expressed as ratios. VEH = saline 1 ml/kg I.P., MMC(7.5) = 7.5 mg/kg mephedrone I.P., MMC(15) = 15 mg/kg mephedrone I.P., MMC(30) = 30 mg/kg mephedrone I.P. All animals were dosed once per day for ten consecutive days followed by a 47 day washout period. No significant differences were found between treatment groups.

## Discussion

Overall, the MMC treatment utilized in these experiments (up to ten daily doses of up to 30 mg/kg MMC I.P.) was well tolerated by all rats. This dosing regimen produced no lethality, no apparent distress and only a very subtle effect on body weight with repeated MMC dosing in Experiment 1 (but not in Experiment 2). The choice of dose was based upon prior work [8] with preliminary observations suggesting that MMC was roughly tenfold less potent than METH in stimulating locomotor hyperactivity. The 30 mg/kg MMC dose in rats may be slightly higher than a standard human single dose (perhaps 200–300 mg), but it is worth noting that typical human use involves multiple doses of MMC consumed in a single session [50], with mean per-session consumption in regular MMC users exceeding one gram [30].

The locomotor response induced by 30 mg/kg MMC confirmed our prior findings of profound hyperactivity with the drug [8]. As with our earlier study, the locomotor stimulation observed surpassed the METH effect in magnitude, even though the dose of METH used here was 2.5 mg/kg rather than the 2 mg/kg used in our previous study. Locomotor hyperactivity with MMC has also been observed with a relatively low dose (3 mg/kg, but not 1 mg/kg S.C.) of the drug in a previous study [9].

There was no sign of behavioral sensitization in Experiment 1, with rats on their tenth daily dose of METH or MMC showing similar distance travelled to those given their very first dose (Figure 2). The absence of behavioral sensitization was matched by an equivalent lack of difference in most neurochemical parameters in rats given single versus repeated dosing (Table 2). Sensitization phenomena with amphetamines and MDMA can be relatively subtle and are influenced by factors such as context, age and strain of animal, dose level and dose intermittency, baseline habituation and the behavioral measure employed [51]. While researchers have frequently found robust sensitization effects in METH treated animals, these findings often utilize dosing regimens substantially in excess of those given in the current study (e.g.: [52], [53]). Sensitization effects are also demonstrated through behavioral measures other than simple distance travelled; for example, Wallace et al. [53] used proportion of time in stereotypy rather than total locomotor behavior. It therefore remains possible that the treatments utilized in the current experiment may elicit some degree of behavioral sensitization that is not captured by simple locomotor measures.

Age factors may also have a substantial effect on behavioral sensitization; while stimulant drug treatment can induce locomotor sensitization in both adult and adolescent rats, these effects are generally less reliable in adolescents [54]. The temporal pattern of drug treatment may have also contributed to the lack of a clear sensitization effect, as a daily schedule of drug treatment (used in the current study) generally produces less sensitization than a pattern involving multiple periods of drug consumption separated by drug-free “holiday” periods [54], [55].

MMC triggered substantial alterations in DA and 5-HT systems in the striatum and the 5-HT system in the hippocampus measured 1 h post dosing. These results are to a certain extent predictable from recent *in vivo* and *in vitro* studies [9], [10], [11] that demonstrate the strong affinity of MMC for DAT and SERT and the modulation of extracellular DA and 5-HT in the nucleus accumbens. Our results, using an *ex vivo* snapshot of regional neurotransmitter content, indicate that at 1 h following 30 mg/kg MMC, whole tissue concentrations of DA were significantly elevated in the striatum while DA metabolism (indexed by measuring HVA+DOPAC/DA) was significantly decreased. In contrast 5-HT levels were significantly decreased by MMC and 5-HT metabolism (indexed by 5-HIAA/5-HT) increased. By comparison, METH had similar effects to MMC on DA and DA metabolism but no significant impact upon 5-HT tissue levels or 5-HT metabolism. Hippocampal 5-HT and 5-HT metabolism effects with MMC were similar to those seen in the striatum.

The contrasting patterns of change seen in DA and 5-HT systems 1 h after MMC dosing (DA increased, DA metabolism decreased; 5-HT decreased, 5-HT metabolism increased) suggests that MMC may trigger a brief but vigorous efflux of 5-HT, thereby inducing the short-lived euphoric rush that characterizes the initial phase of the drug experience [50], [56]. This is in agreement with microdialysis observations of a very rapid and sudden peak in 5-HT levels approximately 20 min after MMC administration [9]. Presumably this 5-HT is then rapidly metabolized to 5-HIAA to produce the pattern of increased 5-HT metabolism and decreased 5-HT levels observed in the present study 1 h after dosing. The dramatic reduction of 5-HT levels following MMC dosing suggests the possibility of exhausting the 5-HT response with closely-spaced multiple doses [10]. This may explain user reports of a substantial weakening of the initial euphoric effect following repeated dosing within a single session [50]. An MDMA-like inhibition of tryptophan hydroxylase could also form part of the explanation for this rapid partial tolerance [57]. As disturbances of 5-HT function have been repeatedly shown to be linked to risk of suicide [58], there may be a connection between this finding and the apparently high levels of suicide amongst MMC-linked fatalities [59].

In contrast, the increase in DA levels may represent a more gradual and sustained effect of the drug. This could be achieved by increased release of DA in some brain areas [9] coupled to an inhibition of DA metabolism and reuptake. A specific inhibition of monoamine-oxidase B (involved in DA breakdown) but not monoamine oxidase A (involved in 5-HT breakdown) by MMC is one plausible explanation of this effect. Cathinone (as well as some of its derivatives) appears to have such an effect, although MMC may not necessarily do so [60], [61].

The dopaminergic effects of MMC may be responsible for the substantial locomotor stimulation, which is frequently reported by users to be of longer duration than the initial euphoric effects of the drug [50]. Again, this agrees with recent findings that MMC induces a rapid and substantial increase in both DA and 5-HT in the nucleus accumbens, but that the 5-HT response is greater in magnitude and shorter in effect [9]. Previous work in this laboratory demonstrated a massive induction of the Fos protein throughout the striatum following a single dose of MMC [8]. The combination of the psychological appeal of the short-lived initial euphoric impact and craving effects associated with activation of the mesolimbic DA pathway provide the foundation for a powerfully addictive drug that is readily self administered by rats via the intravenous route [10].

Interestingly, there was no significant difference in DA or 5-HT levels with single *versus* repeated administration of either drug. However, MMC caused a significantly greater reduction in 5-HT metabolism in the repeated MMC group than the single treatment group, suggesting the induction of subtle neuroadaptations in 5-HT systems with repeated MMC. In addition, the repeated MMC group showed lower HVA and a strong trend towards lower DOPAC than the single group suggesting similar subtle alterations in brain DA metabolism with repeated MMC.

No signs of neuroinflammation were evident with MMC or METH using [^125^I]CLINDE autoradiography. This is in accord with the general absence of overt brain injury noted by other researchers with MMC [23]. While a more rigorous dosing regimen of MMC may induce an inflammatory response, such an approach is likely to require dosing levels well beyond those seen in typical human use. The absence of inflammation in the METH group was not unexpected. Although METH has been clearly shown to be capable of inducing an inflammatory response [40], [41], such a response typically involves doses substantially in excess of those used here, administered in “binge” fashion and sometimes with elevated ambient temperatures.

Experiment 2 was also orientated around determining possible lasting changes in 5-HT and DA following MMC. A recent study [10] reported a persistent decrease in brain 5-HT at 7 days following a binge treatment regimen of MMC (4×10 or 25 mg/kg per day for four days). However, other investigators have failed to find any persistent neurochemical impact of MMC dosing with fairly short duration dosing protocols [12], [23]. Comparable drugs (e.g. MDMA, METH) can induce lasting alterations in a variety of neurotransmitter systems when administered at high dose levels [62]. However, after a 57 day washout, our analysis revealed no significant differences in any neurochemical measures in rats treated with MMC daily for 10 days relative to controls.

The discrepancy between this finding and previous results by a different group [10] may reflect the repeated within-session binge administration used by these authors and the shorter interval after dosing at which 5-HT depletion was assessed (1 week). In addition, the rats used in the earlier study were maintained at an elevated temperature (≥27°C) throughout treatment, which can have a strong influence on stimulant-induced neurotoxicity [63].

In the current study, no lasting behavioral effects of repeated MMC treatment were evident in measures of anxiety (elevated plus maze) and social preference. However, the novel object recognition test did reveal a clear deficit in the 30 mg/kg MMC pre-treated group when tested 36 days after drug treatment. In a recent human study [30] MMC users displayed impaired prose recall compared to controls. At present, the cause of this impairment in recognition memory is unknown, although the strong effect of MMC on hippocampal 5-HT and 5-HT metabolism does suggest the possibility of specific toxicity driven by downstream neuroadaptations occurring in response to this initial perturbation of 5-HT. To our knowledge, the present result represents the first behavioral demonstration in rats of a lasting negative effect of MMC consumption and may provide a useful guide to further research into potential MMC neurotoxicity in both laboratory animals and human users.

In summary, our data confirm powerful acute effects of MMC on monoaminergic neurotransmitter systems. However, 10 days of chronic MMC did not appear to induce any overt injury or lasting alteration in DA or 5-HT systems. Despite this, the impairment of recognition memory observed in high-dose animals more than a month after the cessation of drug provides reason to suspect that MMC may still induce major neuroadaptations, albeit of a subtle nature not detected in the current study. Confirming this finding via replication and pursuing a mechanistic explanation for the observed impairment are obvious priorities for future studies.
